# Amphiphilic diblock copolymer of hydrophilic-functionalized lactone and lactide *via* switchable organocatalytic polymerization†

**DOI:** 10.1039/c7ra12843f

**Published:** 2018-01-09

**Authors:** Jinmei Bai, Xiaoying Tang, Yuan Zhang, Jingjing Lin, Minfeng Li

**Affiliations:** Beijing Key Laboratory of Energy Conversion and Storage Materials, College of Chemistry, Beijing Normal University Beijing 100875 China minfeng_li@bnu.edu.cn; Key Laboratory for Polymeric Composite and Functional Materials of Ministry of Education, School of Chemistry, Sun Yat-sen University Guangzhou 510275 China Linjj36@mail.sysu.edu.cn

## Abstract

Main-chain degradable amphiphilic diblock copolymers composed of a hydrophilic-functionalized polyester and PLA were facilely prepared by one-pot ring-opening polymerization (ROP) *via* actively manipulating the catalytic states of an acid–base catalytic system. The resultant block copolymers showed low critical micelle concentration (CMC) in water and were capable of forming stable micelles with optimal hydrodynamic particle size (average diameter 83 nm) and narrow particle distribution.

## Abstract 2

Main-chain degradable amphiphilic diblock copolymers prepared by one-pot ring-opening polymerization *via* actively manipulating catalytic states of an acid–base catalytic system were capable of forming stable micelle with optimal particle size.

## Graphical abstract

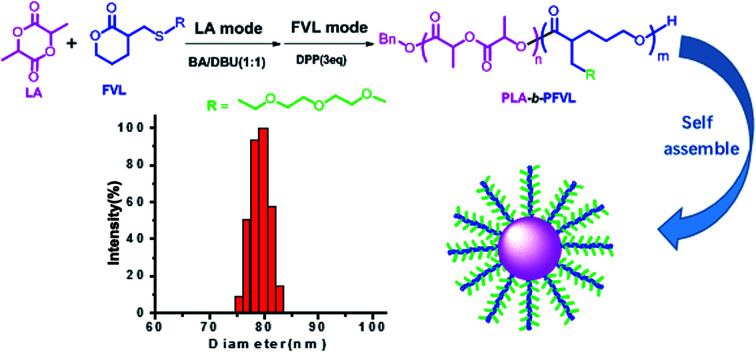

Amphiphilic block copolymers have many important applications in the life sciences, especially in drug delivery. They are able to self-assemble into micelles or polymersomes which are extensively exploited as vehicles for encapsulation and delivery of bioactive reagents. Hydrophobic blocks provided lipophilic compartments or cores for encapsulation of highly hydrophobic therapeutics. Polyesters, especially polylactide (PLA), are widely used as the hydrophobic segments of the copolymers for drug delivery due to their good biodegradability, biocompatibility and renewability.^1^ Hydrophilic blocks form a fully hydrated outer shell for dispersion and reducing bio-molecule attachment. So far, water soluble poly(ethylene glycol) (PEG) is probably the mostly used hydrophilic segment in constructing copolymers for medical applications,^2^ due to its good water solubility and resistance to the adsorption of bio-molecules in plasma. Many PEG-*b*-polyester amphiphilic block copolymers have been synthesized and extensively studied as delivery platforms.^3^ However the inherent non-biodegradable issue of PEGs has caused increasing concerns.^4,5^ Though, high molecular weight PEGs (over 40 kDa) are considered to be metabolically inert, their excretion rates are significantly reduced with increase of molecular weight.^6^ Evidences have emerged showing that high molecular weight PEGs can accumulate and cause vacuolation in the liver, kidney, spleen and tissues after administration.^7,8^ Growing concerns on bioaccumulation and cytoplasmic vacuolization issues of PEG prompted efforts of searching alternatives of PEG. We have recently reported facile preparation of well-defined functional poly(δ-valerolactone) (PFVL) with oligo(ethylene glycol) methyl ether (OEGME) pendant groups and demonstrated that this functional PVL was highly hydrophilic and fully comparable with PEGs in terms of bio-compatibility and capabilities of resistance to non-specific protein adsorption, which made it a promising biomaterial as fully degradable version of PEG for applications in life science.^9^ The excellent protein resistant properties of this hydrophilic polyester led to our further investigation into the feasibility of using PFVL as an alternative of PEG to construct polymeric nano-carriers for drug delivery. As proof of concept, we set out to synthesize well defined amphiphilic diblock copolymers, PFVL-*b*-PLA, with our functional PFVL as the hydrophilic segments and PLA as the hydrophobic blocks, and investigate the self-assembly behaviors of these diblock copolymers.

Conventionally, PEG/polyester copolymers are prepared in a multi-step process which involves isolation and purification of one block before other blocks are installed to the end(s) of the first block by polymerization or by covalent conjugation. This multi-step process is time consuming and more importantly often results in loss of polymer yield and broad polydispersity.^10^ On the other hand, one-pot polymerization methods such as sequential monomer feeding are more efficient and can provide better control over the polymerization processes, which is highly desirable in preparation of copolymers. Though many organometallic catalysts have been successfully used to prepare copolymers by ROP of lactones and lactide,^11,12^ metal-free organocatalysts are generally preferred in preparation of copolymers intended for biomedical applications due to concerns of the possible residual metallic catalyst in polymer product though not all organocatalysts are fully biocompatible.^13,14^ In our previous study, it was found that diphenyl phosphate (DPP) was the most efficient catalyst among other organocatalysts screened for polymerization of δ-valerolactone with oligo(ethylene glycol) functional group under ambient conditions.^9^ Traditional one-pot synthesis of copolymers usually requires a common catalyst that works with high efficiency for all monomers of individual blocks. Unfortunately, in our case, DPP, a weak organic Brønsted acid, works efficient for our functional lactone monomers by electrophilic activation but are almost inactive for lactide which requires basic or nucleophilic activation.^15,16^ Then, we noticed that Hedrick group recently reported a rather surprising discovery that a equimolar mixture of 1,8-diazabicyclo[5.4.0]-undec-7-ene (DBU) and benzoic acid (BA) which was usually used in excess to quench activity of DBU at the end of ROP of lactide turned out to be a better catalyst than DBU itself in terms of promoting well controlled ROP of lactide under mild condition.^17^ It was proposed that an ion pair catalyst system was formed from the mixture of BA and DBU (1 : 1, molar ratio) and a dual-activation mechanism might be involved.^18,19^ Specifically, the protonated DBU cation was believed to be able to activate the initiating/propagating hydroxyl groups and benzoate anion concurrently activate the monomer carbonyl in ROP of LA.^17^ More intriguingly, it was found that the addition of 2 or more equivalents of BA could lead to complete deactivation of this BA/DBU ion pair catalyst system.^17^ Inspired by this discovery, we envisioned that the equimolar BA/DBU ion pair catalyst could be employed to efficiently prepare well defined PLA as the first block then, subsequently, two equivalents of DPP could be added to the reaction mixture to deactivated the BA/DBU ion pair and switch catalytic mode of the catalyst from being “lactide-active” to “lactide-inactive” and “lactone-active”. Thus, the newly added DPP would play double roles: (1) 1 equivalent of DPP would fully deactivate the BA/DBU ion pair (p*K*_a_ of benzoic acid, 4.204, is similar to p*K*_a_ of DPP, 3.88); (2) the other equivalent of DPP would function as catalyst for ROP of functional δ-valerolactones. Herein, we report one-pot preparation of amphiphilic PFVL-*b*-PLA diblock copolymers *via* active switch of activating states of a ROP catalyst complex. The self-assembly of the resultant amphiphilic copolymers in water was then investigated and found them readily form stable micelle structures with ideal particle size in water.

As depicted in Scheme 1, PLA, the hydrophobic segment of the amphiphilic copolymers, can be facilely prepared by well controlled ROP of lactide with BA/DBU (1 : 1) as a bi-functional catalyst. And switching of catalyst states from being lactide active to lactone active is realized by introducing DPP into the reaction mixture. The performance of the catalyst complex in different active states was evaluated by homo-polymerization before employing this catalytic switching strategy in block copolymer preparation. As summarized in Table 1, addition of DPP (1 equiv.) was able to fully quench catalytic activity of BA/DBU in the ROP of lactide (entry 1), which confirmed the hypothesis that DPP could effectively deactivate BA/DBU catalyst, and, as expected, BA/DBU/DPP (1 : 1 : 1) complex showed no catalytic activity towards the functionalized lactone monomers (entry 2). It was postulated that the active sites of both DBU and DPP were mutually deactivated in this state. Increasing the equivalents of DPP to 2, BA/DBU/DPP (1 : 1 : 2), the active state of the complex was expected to be switched and become an efficient catalyst for ROP of the functional lactone. As shown in entry 3, the BA/DBU/DPP (1 : 1 : 2) complex indeed showed capability of promoting ROP of the functional lactone however the polymerization was rather sluggish and proceeded only to 36% conversion in 48 hours, which suggested that the presence of BA/DBU complex greatly compromised the catalytic efficiency of DPP. Further increasing DPP to 3 equivalents, 82% conversion of the functional lactone was reached in 24 hours and homo-polymer obtained had a *M*_n_ close to the theoretically value (based on initiator: monomer ratio) and a PDI value of 1.08 (entry 4). Results from above experiments provided us the optimal acid/base ratio of the catalyst complex in different catalytic mode and clearly indicated that one-pot preparation of PLA-*b*-PFVL block copolymers by tuning the catalytic mode of BA/DBU/DPP based catalyst system was plausible. Next, PLA-*b*-PFVL block copolymers were prepared following the protocol depicted in Scheme 1. Briefly, the ROP of lactide was carried out with benzyl alcohol as initiator and BA/DBU (1 : 1) complex as catalyst and with a [BnOH]_0_/[catalyst]_0_ ratio of 100 : 1 at 36 °C. While lactide conversion reaching 90%, DPP (3 equivalents with regard to BnOH) was introduced to the reaction solution to “turn-off” lactide-active mode of the catalyst complex and switch to lactone-active mode for the ROP of the incoming function lactone monomers. When the conversion of the functional lactone reached 85%, the reaction was quenched by addition of an excess amount of triethyl amine. Following this protocol, block copolymers with various block length were successfully obtained (entry 5–8) with *M*_n_ values close to theoretical ones and fairly narrow molecular weight distribution (PDI, 1.1–1.3, Fig. S2†), which proved that this sequential addition protocol based on facile catalytic mode switching was an efficient strategy for one-pot preparation of block copolymer of lactide and the functional lactone.

**Scheme 1:**
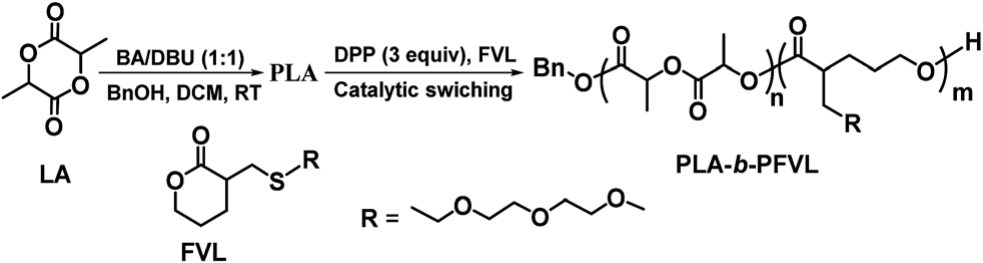
One-pot synthesis of functional lactone-*b*-PLA diblock copolymer through catalytic activity switching.

**Table 1:** Experimental conditions and characterization of the homopolymers and copolymers^a^

Entry	Sample	[DBU]/[BA]/[DPP]	Conv.^d^ (%)	Time	*M*_n theo_ c (g mol^−1^)	*M*_n NMR_ d (g mol^−1^)	*M*_n GPC_ b (g mol^−1^)	PDI^b^
1	PLA	1/1/1	—	48 h	—	—	—	—
2	PFVL	1/1/1	—	24 h	—	—	—	—
3	PFVL	1/1/2	36	48 h	—	—	—	—
4	PFVL	1/1/3	82	24 h	8851	7654	7450	1.08
5	PLA_15_-*b*-PFVL_15_	1/1/3	81	24 h	5450	4742	5032	1.11
6	PLA_20_-*b*-PFVL_30_	1/1/3	81	24 h	9602	8070	7983	1.28
7	PLA_30_-*b*-PFVL_30_	1/1/3	82	24 h	10 467	9456	8694	1.37
8	PLA_50_-*b*-PFVL_50_	1/1/3	82	24 h	17 445	16 777	15 483	1.32
9	PLA_20_-*b*-PFVL_30_	1/1/3	81	24 h	9540	8120	7985	1.26

a Temperature: 36 °C, solvent: CH_2_Cl_2_, [M]_0_ = 0.5 mol L^−1^. Polymerization monomer conversion, time and temperature for the homopolymers or the second block.

b Obtained by GPC in THF.

c Calculated based on *M*_n theo_ = [LA]_0_/[BnOH]_0_ × conv. × (*M*_w_ of LA) + [FVL]_0_/[BnOH]_0_ × conv. × (*M*_w_ of FVL) + (*M*_w_ of BnOH).

d Determined based on ^1^H NMR end-group analysis.

To further demonstrate the high monomer selectivity of the catalyst system in different catalytic mode, polymerizations from mixtures of both monomers were carried out with BnOH as initiator, [M]_0_/[BnOH]_0_/[BA/DBU]_0_ = 20/1/1 at 36 °C. In the first stage, the catalyst mode was set to be lactide-active (*i.e.* BA/DBU (1 : 1) complex) for ROP of lactide only. ^1^H NMR of the reaction mixture revealed that conversion of lactide reached 91% at 48 hours and no conversion of the functional lactone was observed during polymerization of lactide (Fig. 1a and S1† for full spectra). After consumption of lactide monomers, DPP (3 equivalents) was added to the reaction mixture to switch the catalytic mode to being lactone-active and ROP of the functional lactone was initiated. Conversion the functional lactone reached 80% at 24 hours (Fig. 1b). GPC traces showed unimodal and symmetric distribution for both the PLA homopolymer as the first segment and the PLA-*b*-PFVL copolymer indicating the absence of significant prematurely terminated homopolymer or bock copolymer. Moreover, GPC trace of the block copolymers showed a shift towards the higher molar masses indicating initiation and the chain extension of the second segment, PFVL, from the PLA block (Fig. 1c). Thus, the well-defined PLA-*b*-FVL copolymer (PDI 1.26, entry 10) was successfully obtained from mixtures of the two monomers. These results implied that it was highly plausible that facile one-pot preparation of multi-block copolymer from mixtures of monomers with distinct reactivity could be realized by manipulating catalytic modes of a catalyst system.

**Fig. 1:**
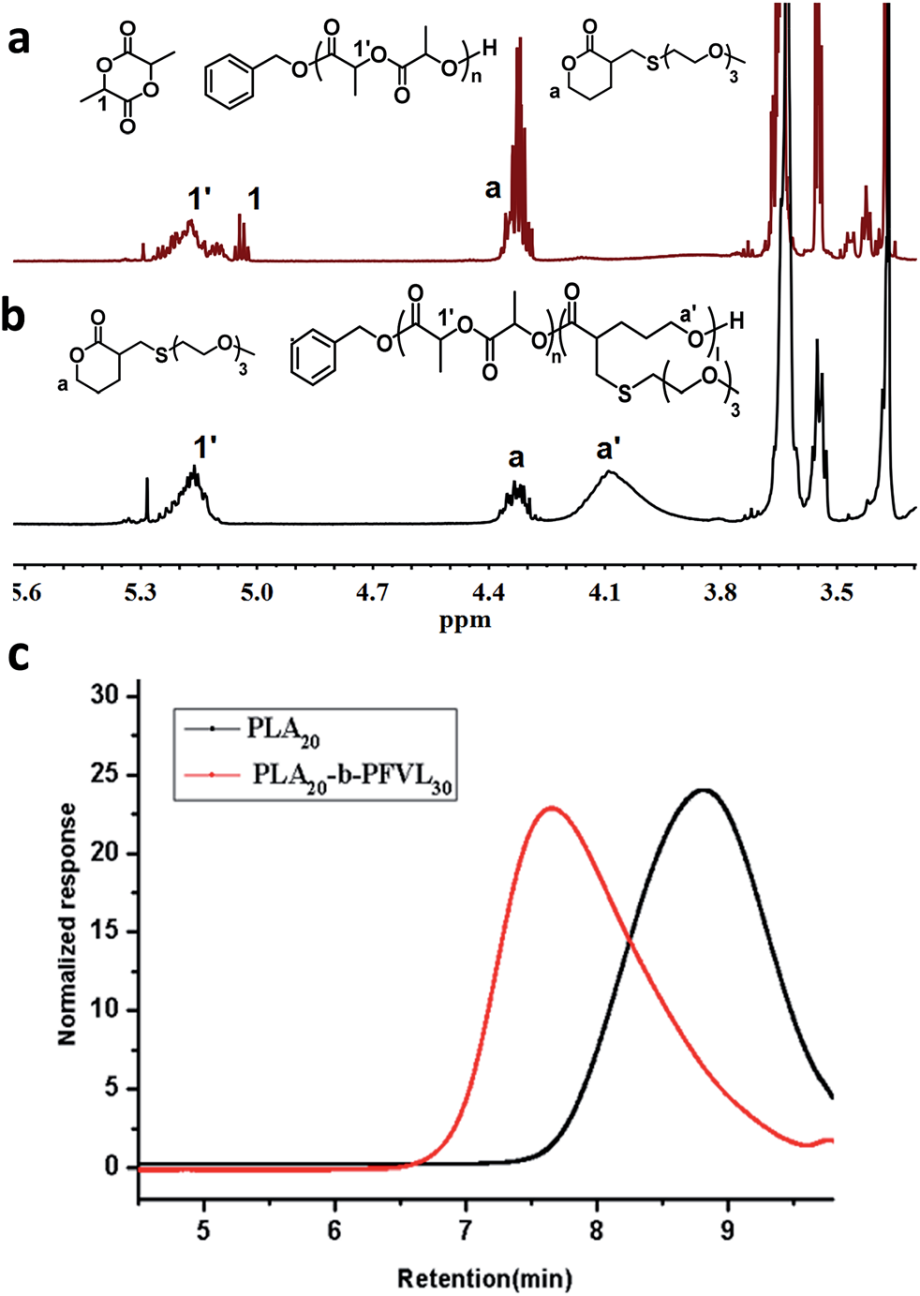
Partial ^1^H NMR of polymerization with mixtures monomers (a) catalyst in lactide-active mold; (b) catalyst state switched to lactone active mold. (c) GPC traces: the red lines correspond to PLA homopolymer as the first block and the black to the PLA-*b*-PFVL copolymer.

Since one of major applications of PEG based amphiphilic copolymers in life science is to be used as nano-carriers of high hydrophobic therapeutics in form of micelle. As possible alternative hydrophilic polymer of PEG, micelle formation property of PFVL based amphiphilic copolymers, PLA-*b*-PFVL was investigated. The critical micelle concentration (CMC) of prepared amphiphilic copolymers, PLA-*b*-FVL, in water was first determined with a pyrene based fluorescence probe assay.^20,21^ The CMC of PLA_20_-*b*-PVF_30_ was found to be around 8.95 mg L^−1^ (Fig. S3†) which was similar to that of MPEG-PLA (8 mg L^−1^) and MPEG-PCL (10 mg L^−1^).^22^ Low CMC is generally considered to be advantageous for drug carriers especially in intravenous applications where high dilution occurs once encapsulated drug entered blood stream. Polymeric micelle was prepared by a thin-film hydration method. Generally, the copolymer was dissolved in acetonitrile and solvent was evaporated leaving a thin film of the copolymer which was dissolved with pure water resulting in micelle solution (see ESI†). Dynamic light scattering (DLS) analysis revealed that micelle formed from PLA_20_-*b*-PVF_30_ had average diameter of 83 nm and rather narrow particle distribution (PDI = 0.12) (Fig. 2a). Scanning electron microscope (SEM) image showed that the micelle appeared to be spherical and particle size was around 75 nm in diameter (Fig. 2b) which was slightly smaller than its hydrodynamic particle size given by DLS analysis due to dehydration during the SEM sample preparation. Furthermore, the particle size and size distribution of the micelle solution was observed to remain almost unchanged for several days at room temperature (Fig. S4†), which suggested that polymeric micelle formed was highly stable under ambient conditions.

**Fig. 2:**
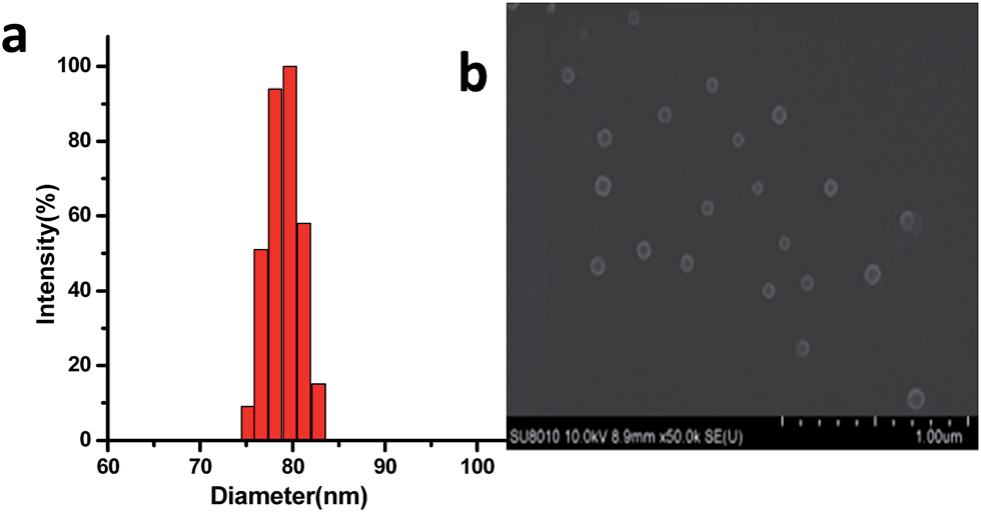
Copolymer PLA_20_-*b*-PVF_30_ in pure water: (a) micelle size distribution of the copolymer analyzed by DLS; (b) SEM image of micelle from the copolymer.

In summary, main chain degradable amphiphilic diblock copolymers were facilely prepared from hydrophilic-functionalized δ-valerolactone and lactide by one-pot ROP which was realized by actively manipulating catalytic states of an acid–base catalytic system. High monomer selectivity of the different catalytic states was demonstrated by successful preparation of PFVL-*b*-PLA copolymer starting with a mixture of two types of monomers, LA and FVL, with distinct reactivity. These results suggested that novel polymerization catalyst systems with multiple distinctive catalytic states might provide facile access to sophisticated polymeric architectures *via* an efficient one-pot polymerization process. The resultant block copolymers showed low critical micelle concentration (CMC) in water and were capable of forming stable micelle with optimal hydrodynamic particle size for drug delivery and narrow particle distribution. Moreover, hydrophilic-functionalized PCL are expected to have relatively rigidified backbone comparing with very flexible PEG chain due to steric repulsion between its hydrophilic side chains, which may lead to deferent surface properties and architectures of nanoparticle (NP) assembled from hydrophilic-functionalized PCL based amphiphilic copolymers in comparison with that of PEG based copolymer. These differences in the physicomechanical aspects of carrier particles may have profound impacts on interactions between NPs and bio-molecules.

## Conflicts of interest

There are no conflicts to declare.

## Supplementary Material

RA-008-C7RA12843F-s001

## References

[cit1] Uhrich K. E., Cannizzaro S. E., Langer R. S., Shakeshwff K. M. (1999). Chem. Rev..

[cit2] Duncan R. (2003). Nat. Rev. Drug Discovery.

[cit3] Maglio G., Nese G., Nuzzo M., Palumbo R. (2004). Macromol. Rapid Commun..

[cit4] Conover C. L. L., Linberg R., Shum K., Shorr R. G. L. (1996). Artif. Cells, Blood Substitutes, Immobilization Biotechnol..

[cit5] Yamaoka T., Tabata Y., Ikada Y. (1994). J. Pharm. Sci..

[cit6] (b) Webster R. , Elliott V., Park B. K., Walker D., Hankin M. and Taupin P., in PEGylated protein drugs: Basic science and clinical applications, Springer, 2009, pp. 127–146

[cit7] Conover C. D., Linberg R., Gilbert C. W., Shum K. L., Shorr R. G. L. (1997). Artif. Organs.

[cit8] Ulery B. D., Nair L. S., Laurencin C. T. (2011). J. Polym. Sci., Part B: Polym. Phys..

[cit9] Li X. W., Li H., Zhao Y. Y., Tang X. Y., Ma S. F., Gong B., Li M. F. (2015). Polym. Chem..

[cit10] Hadjichristidis N. , Pispas S. and Floudas G., Block Copolymers, Wiley, Hoboken, 2003

[cit11] Paul S., Zhu Y. Q., Romain C., Brooks R., Saini P. K., Williams C. K. (2015). Chem. Commun..

[cit12] Dechy-Cabaret O., Martin-Vaca B., Bourissou D. (2004). Chem. Rev..

[cit13] Nachtergael A., Coulembier O., Dubois P., Helevenstein M., Duez P., Blankert B., Mespouille L. (2015). Biomacromolecules.

[cit14] Xia Y., Shen J., Alamri H., Hadjichristidis N., Zhao J., Wang Y., Zhang G. Z. (2017). Biomacromolecules.

[cit15] Makiguchi K., Satoh T., Kakuchi T. (2011). Macromolecules.

[cit16] Makiguchi K., Kikuchi S., Yanai K., Ogasawara Y., Sato S. I., Satoh T., Kakuchi T. (2014). J. Polym. Sci., Part A: Polym. Chem..

[cit17] Coagy D. J., Fukushima K., Horn H. W., Rice J. E., Hedrick J. L. (2011). Chem. Commun..

[cit18] Coulembier O., Sanders D. P., Nelson A., Hollenbeck A. N., Horn H. W., Rice J. E., Fujiwara M., Dubois P., Hedrick J. L. (2009). Angew. Chem., Int. Ed..

[cit19] Dove A. P., Pratt R. C., Lohmeijer B. G. G., Waymouth R. M., Hedrick J. L. (2005). J. Am. Chem. Soc..

[cit20] Kalyanasundaram K., Thomas J. K. (1977). J. Am. Chem. Soc..

[cit21] Aguiar J., Carpena P., Molina-Bolívar J. A., Carnero Ruiz C. (2003). J. Colloid Interface Sci..

[cit22] Chena L. J., Tana L. W., Zhangb X. N., Lib J., Qiana Z. Y., Xianga M. L., Wei Y. Q. (2015). Int. J. Pharm..

