# Supplementary material for: Amphiphilic diblock copolymer of hydrophilic-functionalized lactone and lactide via switchable organocatalytic polymerization
Source: RSC Adv. 2018 Jan 9;8(4):1905–8. doi: 10.1039/c7ra12843f (PMC9077283; doi:10.1039/c7ra12843f)
Supplement: RA-008-C7RA12843F-s001 [file RA-008-C7RA12843F-s001.pdf]

Electronic Supplementary Material (ESI) for RSC Advances.
This journal is © The Royal Society of Chemistry 2017

Supporting Information

Amphiphilic Diblock Copolymer of Hydrophilic-Functionalized Lactone and Lactide via Switchable Organocatalytic Polymerization

Jinmei Bai,[a] Xiaoying Tang,[a] Yuan Zhang,[a] Jingjing Lin,[b]* Minfeng Li[a]*

[a]Beijing Key Laboratory of Energy Conversion and Storage Materials, College of Chemistry, Beijing Normal University, Beijing 100875, China.

[b]Key Laboratory for Polymeric Composite and Functional Materials of Ministry of Education, School of Chemistry, Sun Yat-sen University, Guangzhou 510275, China.

Table of Contents

1. Materials and Methods S2

2. General preparation procedures of homopolymers S3

3. General preparation procedures of block copolymers S3

4. Examples of ^{1}H NMR Analysis S4

5. Examples of GPC Analysis S7

6. Critical micelle concentration (CMC) measurement S9

7. Micelle preparation S10

8.The result of DLS S10

9.SEM measurements S10

10. References S11

1. Materials and Methods

Chemicals were purchased from commercial sources and used as received unless otherwise noted. The functional δ-valerolactone was synthesized according to reported procedures.[1] Both lactide and the functional δ-valerolactone used in this study were racemic mixtures. The catalyst DBU-BA was synthesized according to reported procedures.[2] Silica gelfor analytical thin layer chromatography (TLC) and column chromatography (200~300 mesh) were purchased from Qingdao Haiyang Chemical Co., Ltd & SpecialSilica Gel Factory (Qingdao, China). Dialysis was carried out with tubing cellulose membrane (MWCO 1KD, Spectrum Laboratories Inc. USA). The ^{1}H NMR spectra were recorded at 400 MHzon a Bruker AV400 spectrometer atambient temperature. Chemical shifts were reported in parts per million (ppm)downfield from TMS (tetramethylsilane). Gel Permeation Chromatography (GPC) in THF was performed using three Agilent PL-gel columns (Mixed C, Mixed C, and Mixed D), a Waters 1515 isocratic HPLC pump, and a Waters2414 RI detector. THF was used as the eluent at a flow rate of 1.0 mL/min at 35℃. Polystyrenestandards (American Polymer StandardsCorp, USA) were used for the calibration.Particle size distributions of the samplesdispersed in water were measured with dynamic light scattering (DLS) (Zeta PLUS, Brookhaven Instruments Corporation, NY, USA). The DLS instrument was operatedunder the following conditions: temperature 25 °C, detectorangle 90°, incident laser wavelength 658 nm. SEM images were obtained by a field emission scanning electron microscopy (FESEM, S-8010，Hitachi).

2. General preparation procedures of homopolymers

The functional δ-valerolactones (2.19 g, 7.50 mmol), benzyl alcohol (0.05 g, 0.50 mmol), catalyst DBU-BA (0.27 g, 1.00 mmol) and diphenyl phosphate (0.75 g, 3.00 mmol) in 1.5 mL DCM was added to a 25 ml flask equipped with a rubber septum connected to N2 vacuum line. Moisture and oxygen were removed by freeze-thawing protocol. The polymerization was carried out at room temperature under protection of nitrogen. The monomer conversion was monitored by analyzing aliquots of reaction mixture with ^{1}H NMR. Upon completion, the reaction mixture was quenched by addition of excess amount of triethylamine, solvent was evaporated under vacuum and residue was dissolved in methanol, then transferred into a dialysis bag (MWCO = 1000) with methanol as dialysis solvent. The solvent was replaced every 12 hours for 3 times. Then the methanol solution was concentrated and the residual solvent was removed via high vacuum pump for 12 h.

3. General preparation procedures of block copolymers

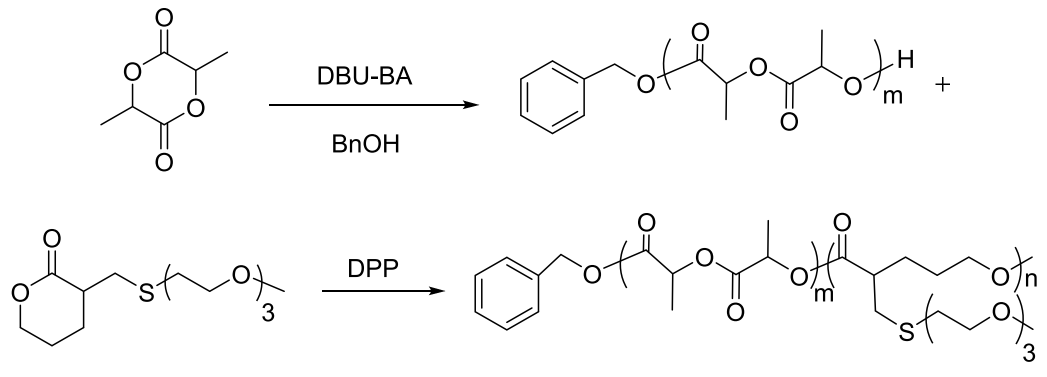

Lactide (1.08 g, 7.50 mmol) was added to a 25 ml flask equipped with a rubber septum connected to N2 vacuum line. Benzyl alcohol (0.05 g, 0.50 mmol) and catalyst DBU-BA (0.27g, 1.00 mmol) were dissolved in DCM and added to the flask. Moisture and oxygen were removed by freeze-thawing protocol. The polymerization was carried out at room temperature under protection of nitrogen. The conversion of the lactide was monitored by analyzing aliquots of reaction mixture with ^{1}H NMR.

Upon completion of the PLA block, diphenyl phosphate (0.75 g, 3.00 mmol) in DCM was introduced by a syringe. Before addition of monomers of the second block, thee reaction mixture was stirred for 5 min. The functional δ-valerolactones (2.19 g, 7.50 mmol) in DCM was introduced by syringe. The monomer conversion was monitored by analyzing aliquots of reaction mixture with ^{1}H NMR. Upon completion, the reaction mixture was quenched by addition of excess amount of triethylamine, solvent was evaporated under vacuum and residue was dissolved in methanol, then transferred into a dialysis bag (MWCO = 1000) with methanol as dialysis solvent. The solvent was replaced every 12 hours for 3 times. Then the methanol solution was concentrated and the residual solvent was removed via high vacuum pump for 12 h.

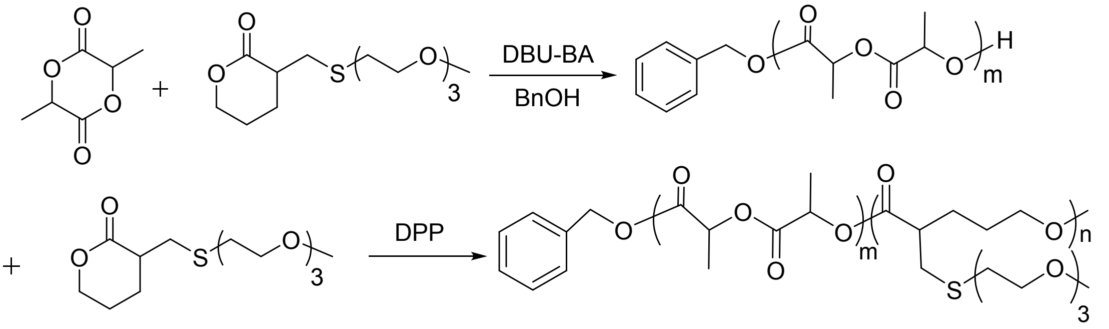

For the one-pot approach, Lactide (0.72 g, 5.00 mmol) and the functional δ-valerolactones (2.19 g, 7.50 mmol) were added to a 25 mL flask equipped with a rubber septum connected to N2 vacuum line at the beginning. Benzyl alcohol (0.05 g, 0.50 mmol) and catalyst DBU-BA (0.27g, 1.00 mmol) were dissolved in DCM and added to the flask. Moisture and oxygen were removed by freeze-thawing cycles. The polymerization was carried out at room temperature under protection of nitrogen. The conversion of the lactide was monitored by analyzing aliquots of reaction mixture with ^{1}H NMR. Upon completion of the PLA block, diphenyl phosphate (0.75 g, 3.00 mmol) in DCM was introduced by a syringe. Upon completion, the reaction mixture was quenched by addition of excess amount of triethylamine, solvent was evaporated under vacuum and residue was dissolved in methanol, then transferred into a dialysis bag (MWCO = 1000) with methanol as dialysis solvent. The solvent was replaced every 12 hours for 3 times. Then the methanol solution was concentrated and the residual solvent was removed via high vacuum pump for 12 h.

4. Examples of ^{1}H NMR Analysis

a

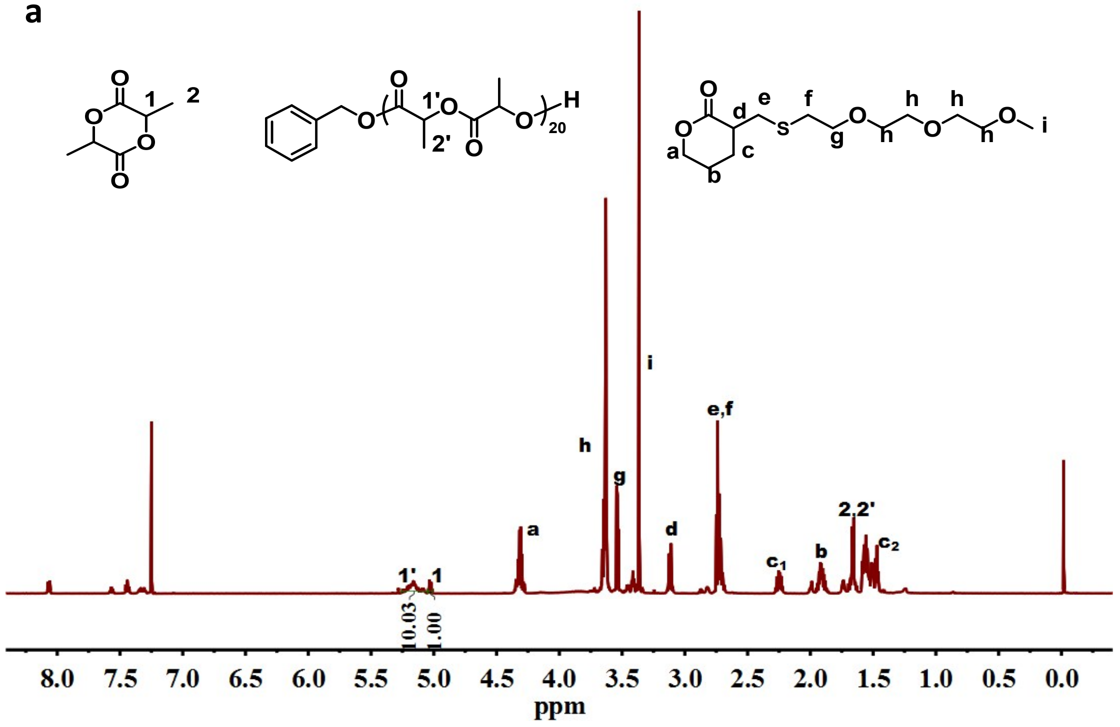

b

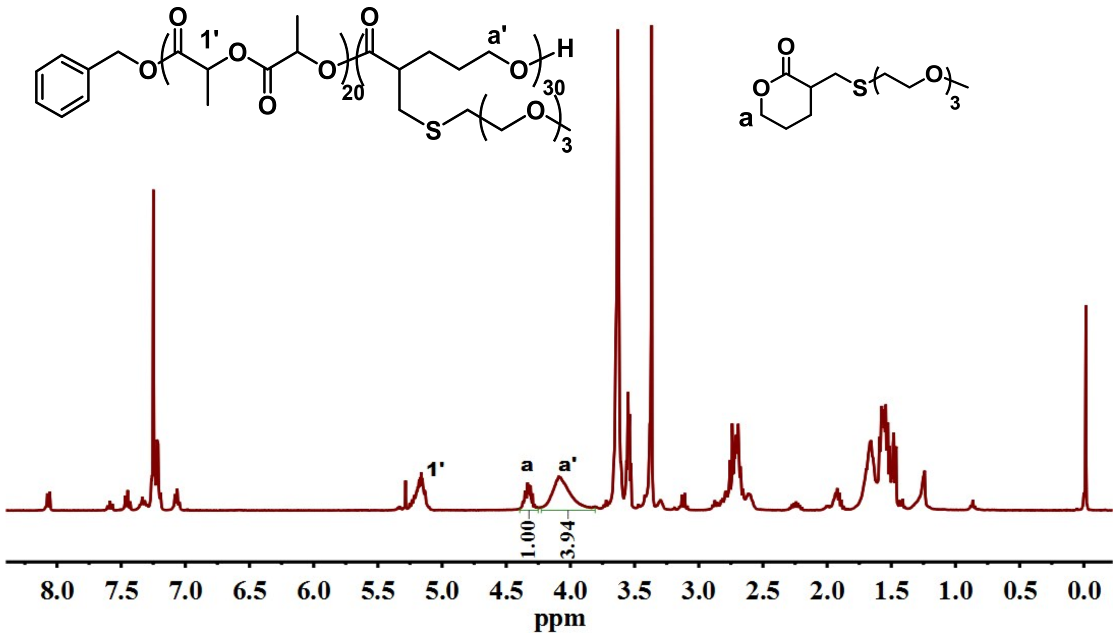

c

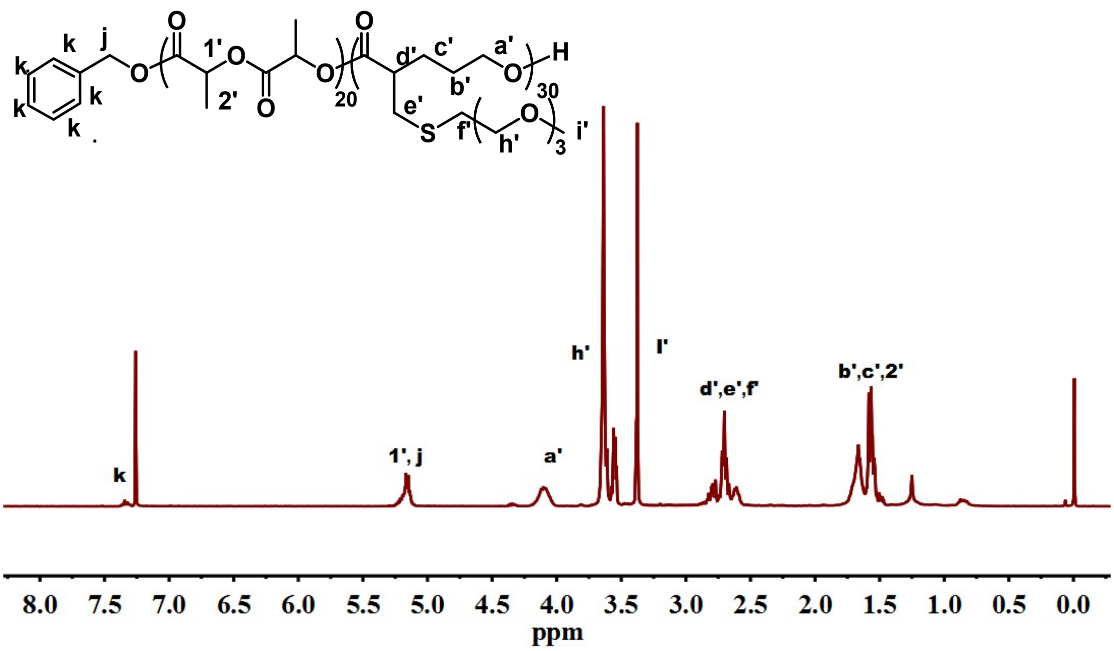

Figure S1. Full ^{1}H NMR of polymerization with mixtures monomers (a) catalyst in lactide-active mold; (b) catalyst state switched to lactone active mold; (c) ^{1}H NMR spectra of purified PLA$_{20}$-*b*-PFVL$_{30}$ prepared by the one-pot method starting with mixture of LA and FVL.

5. Examples of GPC Analysis

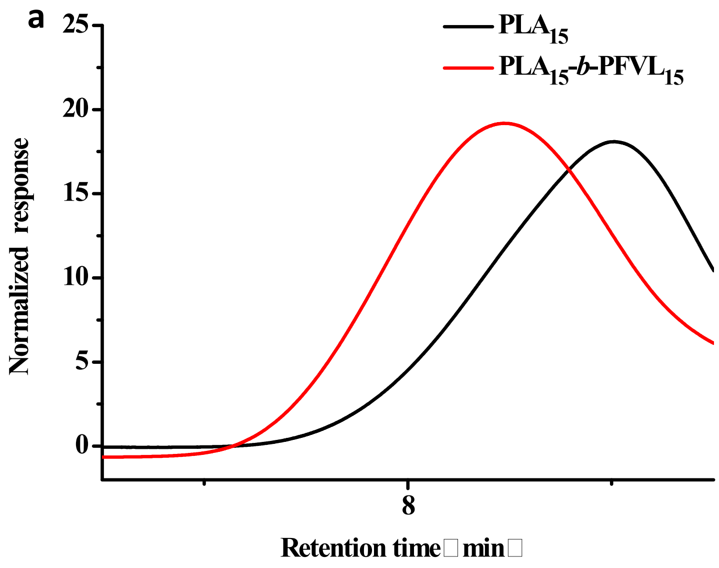

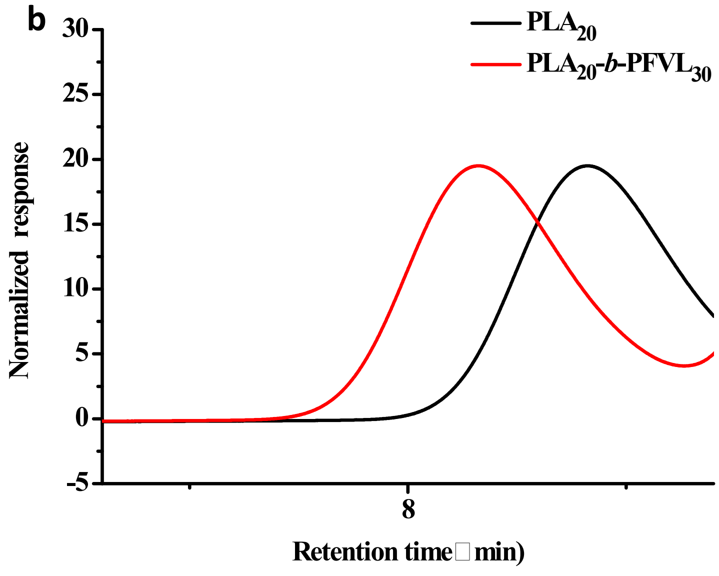

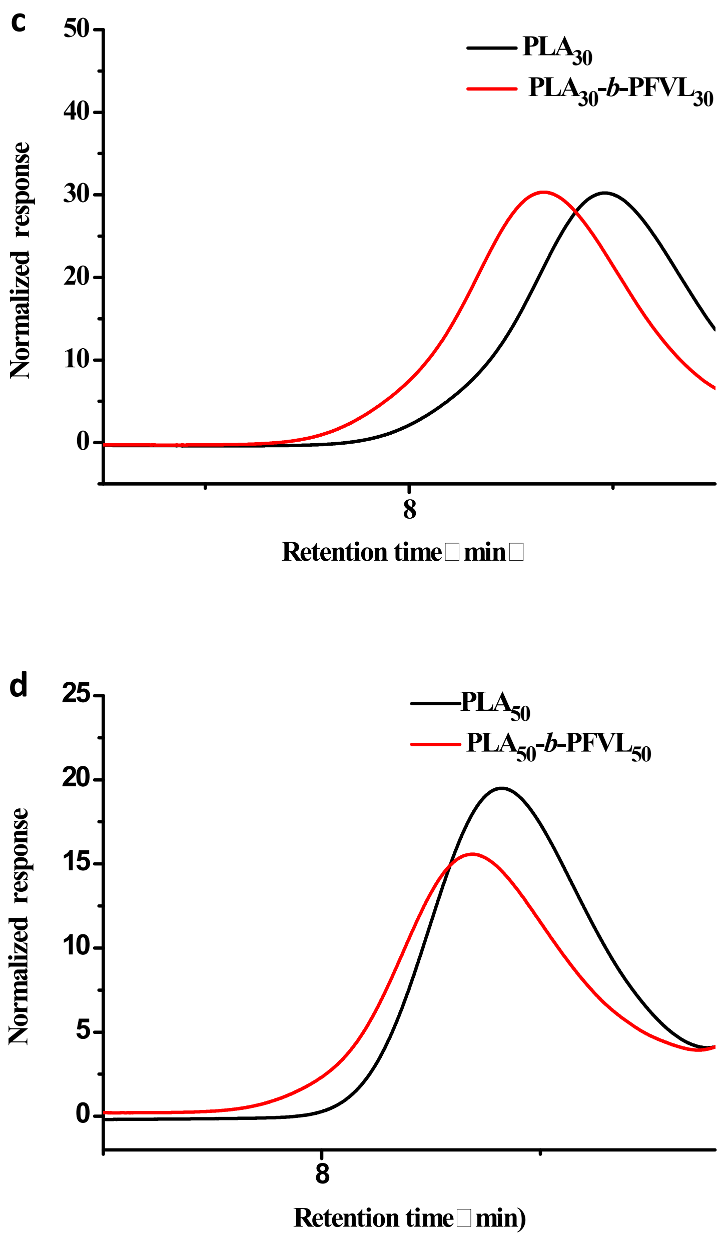

Figure S2. GPC traces of polymers during sequential feeding, the black lines correspond to PLA homopolymer as the first block and the black to the PLA-*b*-PFVL copolymer.: (a) **PLA$_{15}$-*b*-PFVL$_{15}$**, (b) **PLA$_{20}$-*b*-PFVL$_{30}$**, (c) **PLA$_{30}$-*b*-PFVL$_{30}$** and (d) **PLA$_{50}$-*b*-PFVL$_{50}$**.

6. Critical micelle concentration (CMC) measurement

The CMC of the copolymerwas measured using pyrene as a fluorescent probe.[3] 10.14 mg (0.05mmol) pyrene was dissolved in acetone and diluted to 250 mL with a volumetric flask. 0.1 mL of a pyrene stock solution was evaporated in a 10-mL volumetric flask under room temperature overnight. Then 10 mL aqueous solution with concentration in the range from 10.4 mg/L to 130.0 mg/L were added to each pyrene-containing volumetric flask respectively. Then the solutions were allowed to standat room temperature for 24 h. Then the spectra were recorded in the 340–500 nm wavelength range with an excitation wavelength of 339 nm. The emission of the first (I_1, 373 nm) and third (I_3, 384 nm) vibronic bands were measured. All fluorescence measurementswere carried out at 25.0 ^{0}C.The CMC wasgiven by the copolymer concentration ofthe inflection point in the plot of pyrene I_1/I_3 ratio as a function of the concentration of the copolymer.

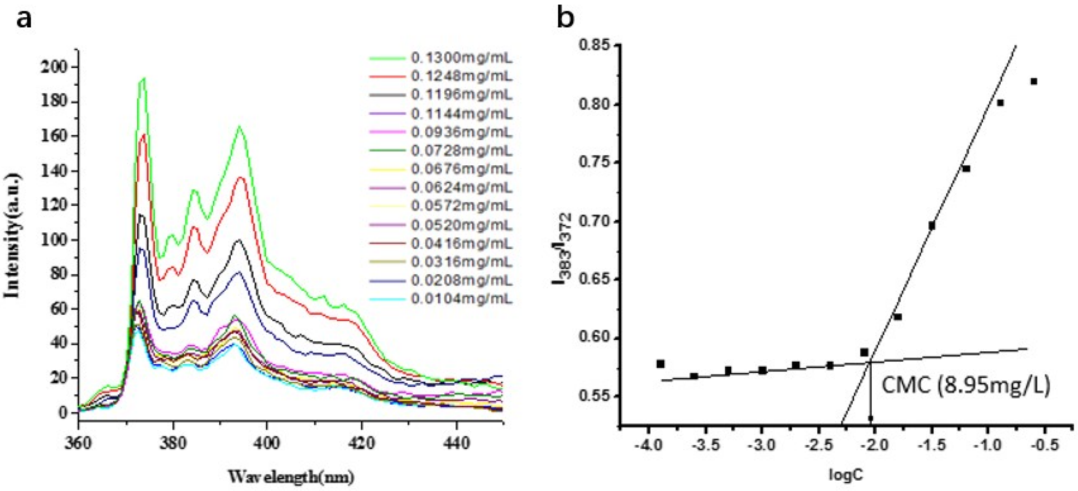

Figure S3. Determination of CMC of **PLA$_{20}$-*b*-PFVL$_{30}$:** (a) Fluorescenceemission of pyrene as a function of the concentration of the copolymer; (b) Plots of pyrene I_1/I_3ratio as a function of the concentration of the copolymer.

7. Micelle preparation

1 mg diblock polymer obtained from evaporation of 60 μL stock solution (16.6 mg/L in methylene chloride) was dissolved with 5 mL acetonitrile in a 25 mL round bottom flask. Acetonitrile was then removed by rotary evaporation at 40 ℃*in vacuo* and further dried under high vacuum overnight to form a transparent film on the wall of the flask. Then the resulting polymer film was fully dissolved with 5 mL of ultrapure water at 60 ℃ to form a clear micelle solution which were filtered through a 0.45 μm membrane before DLS analysis.

8. The result of DLS

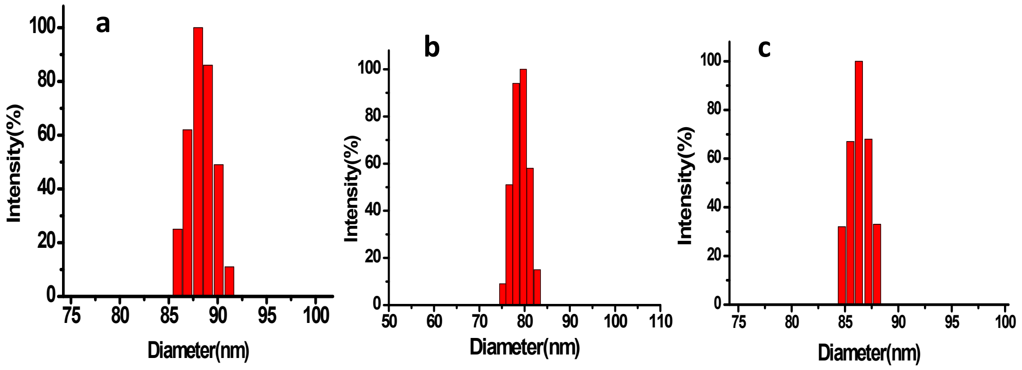

Figure S4. Size distribution of **PLA$_{20}$-*b*-PFVL$_{30}$**in 1(a), 8 (b) and 20 days (c).

9. SEM measurements

For SEM, 10 μL of micelle solution was deposited on a clean aluminumfoil, and water is allowed to evaporate. A thin layer of gold is coated onto sample prior imaged with a Hitachi(FESEM, S-8010) SEM at an accelerating voltage of 10 kV.

10. References

1.X. W. Li, H. Li, Y. Y. Zhao, X. Y. Tang, S. F. Ma, B. G, and M. F. Li, *Polym. Chem.*, 2015, **6**, 6452.

2.D. J. Coagy, K. Fukushima, H. W. Horn, J. E. Rice, and J. L. Hedrick, *Chem. Commun.,*2011, **47**, 3105.

3.J. Aguiar, P. Carpena, J. A. Molina-Bolívar, and C. Carnero Ruiz, *Journal of Colloid and Interface Science*, 2003, **258**, 116.
